# Barriers to Accessing Antiretroviral Treatment Among Key Populations in Southwest Nigeria

**DOI:** 10.7759/cureus.59312

**Published:** 2024-04-29

**Authors:** Prosper Okonkwo, Olaposi J Olatoregun, Olatunbosun Abolarin, Olasunkanmi Olajide

**Affiliations:** 1 Public Health, APIN Public Health Initiatives, Abuja, NGA

**Keywords:** health system challenges, nigeria, barriers to access, key populations, antiretroviral therapy, hiv/aids

## Abstract

Background

In Nigeria, similar to most of sub-Saharan Africa, the fight against HIV/AIDS is hampered by various barriers. Particularly, key populations (KP) face unique challenges in accessing antiretroviral therapy (ART), attributed to health systems, patient-related, and community-related factors. This study aims to explore these barriers among KP in Nigeria, enhancing understanding to improve ART access and outcomes, thereby contributing to global efforts to end the HIV/AIDS epidemic.

Objectives

The objective of this study is to explore barriers to accessing ART services among KP living with HIV in the southwest region of Nigeria.

Materials and methods

This descriptive cross-sectional study, which was carried out in the southwestern Nigerian states of Ondo and Ogun, involved 4,253 KP attending ART clinics. Using a multistage sampling technique, 219 females and 154 males between the ages of 17 and 72 were chosen. Semi-structured survey questions were used to get information to elicit these barriers. SPSS Statistics (version 29.0; IBM Corp., Armonk, NY) was used for quantitative data analysis.

Results

Specifically, 48.3% of respondents were men, and 58.7% were women; 54.0% were female sexual workers, and the next highest percentages were men who have sex with men (27.0%) and injectable drug users (19.3%); and 79% (51.0% agreed, 28.0% strongly agreed) concurred that the barriers to accessing ART are transportation, distance, and financial challenges. Patients at ART clinics were generally satisfied with the healthcare and health workers' attitude. Measures to protect privacy and confidentiality were considered crucial by the respondents. There are significant associations between ART accessibility and socio-demographic and socio-economic characteristics (p-value <0.05).

Conclusion

This study probed the complex landscape of barriers to ART access faced by KP in Nigeria, underscoring the critical need for tailored, innovative strategies to overcome these obstacles and offering actionable insights for stakeholders to enhance ART adherence and access thereby significantly improving the quality of life for people living with HIV.

## Introduction

HIV/AIDS remains a major public health challenge for populations in both developed and developing countries, with approximately 39 million people living with HIV and 1.3 million new infections in 2022 [1]. The battle against HIV/AIDS in Nigeria, like in many parts of Sub-Saharan Africa, remains a significant public health intervention [2].

Despite advancements in antiretroviral therapy (ART) that have transformed HIV/AIDS from a fatal disease to a manageable chronic condition, thereby improving patients’ outcomes. Certain subpopulations, such as orphans and vulnerable children (OVC), adolescents, and key populations (KP), continue to lag behind due to reasons like poor adherence, treatment compliance, and socio-economic factors [3,4]. Sometimes some of these reasons are not well-defined and understood, especially in KP, and these might make them face barriers in accessing these lifesaving medications. KP, including men who have sex with men (MSM), female sex workers (FSW), people who inject drugs (PWID), and transgenders (TG), bear a disproportionately higher burden of HIV compared to the general population [5]. These groups face unique barriers in accessing ART, which are crucial for stakeholders to combat for Nigeria to achieve the global goals of reducing HIV transmission and improving the quality of life for people living with HIV (PLHIV) [2,6-10].

Various studies have identified and characterized a range of barriers to accessing HIV/AIDS treatment services for PLHIV in Nigeria, including KP [10-13]. Abubakar Saleh categorized these barriers to ART treatment into three main groups: health system-related, patient-related, and community-related barriers [13]. Health system-related barriers include poor treatment literacy, long waiting times, inadequate health infrastructure, limited availability of ART medications, and the facility not implementing person-centred or integrated approach [8,9]. Patient-related barriers encompass factors such as denial of HIV status, socioeconomic status, transportation, poor awareness, fear of adverse drug effects, and the stigma associated with HIV/AIDS [7]. Community-related obstacles include the criminalization of same-sex relationships by the Nigerian government, societal stigma, and discrimination against KPs, which can deter them from seeking treatment [14,15].

Innovative approaches to KP programming, such as "one-stop shop" (OSS), moonlight models, and KP hotspot mapping, have been implemented to improve access to ART among KPs and to implement KP-specific interventions [16,17]. The most widely implemented is the OSS, which aims to provide integrated healthcare services in an environment devoid of stigma, thereby promoting retention and ART linkage [17]. However, the high attrition and presence of drug resistance among KP groups mean more has to be done to improve patient outcomes in KP [2,6].

This study therefore explored barriers to accessing ART services among KP living with HIV in Nigeria. By understanding and addressing these barriers, stakeholders can work towards improving ART access, adherence, and outcomes among KP, ultimately contributing to the global goal of ending the HIV/AIDS epidemic.

## Materials and methods

Study settings

This study was conducted in the Ogun and Ondo states of southwest Nigeria, which were purposefully selected as states with established ART infrastructure. The study encompassed KP people living with HIV (KPLHIV) aged 18 years and older who were actively enrolled in the ART programs through the OSS, having been under ART for a minimum duration of three months and self-identifying as KP categories of PWID, MSM, FSWs, TG, and people in prisons and other enclosed settings. This criterion ensured the inclusion of participants with sufficient exposure to ART.

Sample size determination

Multistage sampling was used in this study. Ogun and Ondo were the two states purposefully selected out of six states in the southwest. There is one OSS in each of the states, and both OSSs were selected. A sample size of 373 was determined using the Cochran formula, and it was assigned to the different KP types using the probability proportional to size (PPS) method. Cronbach's alpha gave a reliability score of 0.82. A total of 4,253 KP in the states of Ondo and Ogun who were currently receiving ART made up the study population. Of this number, 373 participants (219 women and 154 men) were selected through multistage sampling methods. They fulfilled the inclusion requirements of being members of the KP, living with HIV, and being on ART for a minimum of three months.

Data collection

Data were collected from January 16 to January 24, 2024, using a semi-structured questionnaire, which was initially tested and refined through a pilot study in an OSS in Abuja to ensure reliability. The open data kit (ODK) platform was used for data entry. Research assistants underwent two weeks of training focusing on study goals, research methodology, and ethical considerations. Data security was prioritized, with encryption and password protection of stored information on tablets and laptops used. Informed consent was obtained from all participants, who were fully briefed on the study's intent. Confidentiality and participant understanding were emphasized throughout the research process.

Data analysis

The ODK server's data were cleaned and verified in Microsoft Excel 2019 (Microsoft® Corp., Redmond, WA). While qualitative data were subjected to NVivo thematic analysis (QSR International, Melbourne, Australia), quantitative data were subjected to univariate, bivariate, and multivariate analyses. Quantitative data were analyzed on SPSS Statistics (version 29.0; IBM Corp., Armonk, NY). Descriptive statistics were used to examine access, self-reported adherence, and demographic and socioeconomic traits. Logistic regression was used to determine the barriers to ART access.

Ethical consideration

Ethical approval was provided by the National Health Research Ethics Committee (NHREC) with NHREC approval number NHREC/01/01/2007-05/01/2024. Confidentiality and privacy for participants were guaranteed by informed consent. A comprehensive consent form that prioritized data de-identification and confidentiality during analysis and reporting was administered to the participants.

## Results

We explored the descriptive analysis of the socio-demographic and socio-economic characteristics of KPLHIV in the participating OSS. The demographic analysis (Table 1) of the study participants revealed a gender distribution of 41.3% male (n = 154) and 58.7% female (n = 219). The age distribution was concentrated in the ranges of 22-26 years (22.5%), 27-31 years (25.5%), and 17-21 years (16.1%). The population consisted predominantly of FSWs, accounting for 50.4% of participants, followed by MSMs at 27.1%, PWIDs at 19.3%, and TG and those in prisons or other enclosed settings, representing 1.3% and 1.9%, respectively. Regarding marital status, a significant majority (81.0%) were single, with married participants constituting 15.0%, and smaller percentages reported as separated (2.7%), divorced (0.5%), and cohabiting (0.3%). Educational status showed that 55.2% of respondents completed secondary education, 30.0% had tertiary education, 12.3% finished primary education, and 2.4% had no formal education. Employment status among the participants revealed that 39.7% were unemployed, 21.2% were self-employed, 20.1% were students, and 17.7% were employed, with retirees and others comprising 1.3% of the surveyed KPLHIV.

**Table 1:** Descriptive analysis of socio-demographic and socio-economic characteristics of the study participants KPLHIV: Key population people living with HIV; FSW: Female sex workers; PWID: Persons who inject drugs; MSM: Men having sex with men; TG: Transgender

Variables	Categories	Frequency	Percentage (%)
Socio-demographic characteristics of participants			
Gender	Female	219	58.7
	Male	154	41.3
Age Group	17-21	60	16.1
	22-26	84	22.5
	27-31	95	25.5
	32-36	55	14.8
	37-41	33	8.9
	42-46	21	5.6
	47-51	9	2.4
	52-56	9	2.4
	57-61	2	0.5
	62-66	3	0.8
	67-71	1	0.3
	72 above	1	0.3
Client Typology	FSW	188	50.4
	PWID	72	19.3
	MSM	101	27.1
	TG	5	1.3
	People in prisons and other enclosed settings	7	1.9
Marital Status	Widow/widower	2	0.5
	Single	302	81.0
	Married	56	15.0
	Separated	10	2.7
	Divorced	2	0.5
	Cohabiting	1	0.3
Socio-Economic Parameters of KPLHIVs			
Education	None	9	2.4
	Primary	46	12.3
	Secondary	206	55.2
	Tertiary	112	30.0
Employment Status			
	Student	75	20.1
	Self-employed	79	21.2
	Unemployed	148	39.7
	Employed	66	17.7
	Others	5	1.3

Analysis regarding the affordability and accessibility of ART services (Table 2) among KPLHIVs in the surveyed states revealed that approximately 51.5% of respondents acknowledged difficulties in affording transportation to ART clinics, with 28.4% expressing strong agreement. In contrast, a minority (6.2%) strongly disagreed, highlighting that the majority perceived transportation affordability as a major barrier. The mean response score of 3.11, along with a standard deviation (SD) of 1.2, suggests a moderate consensus among participants regarding the financial challenges associated with accessing ART services.

**Table 2:** Descriptive analysis of KPLHIV responses based on availability, affordability, and accessibility ±SD: Standard deviation; ART: Antiretroviral therapy; KPLHIV: Key population people living with HIV

Questions	Responses	Frequency	Percentage (%)	Mean	±SD
I struggle to afford transportation to an ART clinic.	Neutral	26	7.0	3.11	±1.2
	Strongly disagree	23	6.2	-	-
	Disagree	26	7.0	-	-
	Strongly agree	106	28.4	-	-
	Agree	192	51.5	-	-
	Total	373	100.0	-	-
Service charges from my facility impeded me from accessing ART.	Neutral	3	0.8	1.41	±0.7
	Strongly disagree	245	65.7	-	-
	Disagree	109	29.2	-	-
	Strongly agree	6	1.6	-	-
	Agree	10	2.7	-	-
	Total	373	100.0	-	-
I am satisfied with the quality of service provided at my ART clinic.	Neutral	2	0.5	3.47	±0.6
	Strongly disagree	4	1.1	-	-
	Disagree	4	1.1	-	-
	Strongly agree	173	46.4	-	-
	Agree	190	50.9	-	-
	Total	373	100.0	-	-

The study further explored the effect of service fees at ART facilities on PLHIV's ability to receive treatment. While the majority of participants agreed on transportation affordability as a major barrier, only a minimal fraction of participants (2.7% agreed, 1.6% strongly agreed) identified service fees as a barrier to ART access. However, the overwhelming majority (65.7% strongly disagreed, 29.2% disagreed) refuted the notion that service charges deterred them from accessing ART (mean = 1.4, SD = 0.7). This reflects a consensus that, while transportation affordability is a barrier to ART services, service fees at ART clinics do not constitute a significant barrier to treatment access. From our knowledge of ART clinics in Nigeria, the program is donor-funded, and, while there might be service charges in a few cases, it will be minimal and should not affect ART services.

Participant satisfaction with the level of service provided at ART clinics was also assessed, revealing high levels of contentment. A vast majority of respondents (46.4% strongly agree, 50.9% agree) expressed satisfaction with the care received (mean = 3.47, SD = 0.6). This indicates a strong, positive perception of the quality of care at ART clinics among PLHIV, with responses showing low variability and a clear tendency towards agreement.

The analysis of the acceptability (Table 3) of ART services among KPLHIV revealed significant insights. The majority of respondents, comprising 58.4% agreeing and 34.6% strongly agreeing, indicated they were comfortable interacting with medical professionals during ART visits. Conversely, a minority of participants expressed disagreement (20.44%) or strong disagreement (0.55%), with 4.0% remaining neutral. Privacy and confidentiality measures within ART services received a positive mean score of 3.43, reflecting generally favourable regard by participants.

**Table 3:** Descriptive analysis of KPLHIV based on acceptability ±SD: Standard deviation; ART: Antiretroviral therapy; KPLHIV: Key population people living with HIV

Questions	Responses	Frequency	Percentage (%)	Mean	±SD
I feel comfortable discussing my health concerns with healthcare providers at the ART clinic.	Neutral	15	4.0	3.43	±0.900
	Strongly disagree	2	0.5	-	-
	Disagree	9	2.4	-	-
	Strongly agree	129	34.6	-	-
	Agree	218	58.4	-	-
	Total	373	100.0	-	-
Confidentiality and privacy are very important at the ART clinic.	Neutral	14	3.8	3.37	±0.844
	Strongly disagree	1	0.3	-	-
	Disagree	2	0.5	-	-
	Strongly agree	171	45.8	-	-
	Agree	185	49.6	-	-
	Total	373	100.0	-	-
The attitude of staff at my ART clinic has been encouraging.	Neutral	5	1.3	3.46	±0.678
	Strongly Disagree	2	0.5	-	-
	Disagree	3	0.8	-	-
	Strongly Agree	168	45.0	-	-
	Agree	195	52.3	-	-
	Total	373	100.0	-	-
The clinic environment is conducive to the provision of ART services.	Neutral	11	2.9	3.38	±0.809
	Strongly disagree	1	0.3	-	-
	Disagree	9	2.4	-	-
	strongly agree	168	45.0	-	-
	agree	184	49.3	-	-
	Total	373	100.0	-	-
The effects of ART on my health have been bearable with minimal adverse effects.	Neutral	15	4.0	3.38	±0.809
	Strongly disagree	2	0.5	-	-
	Disagree	9	2.4	-	-
	Strongly agree	114	30.6	-	-
	Agree	233	62.5	-	-
	Total	373	100.0	-	-

Staff attitudes at ART centres were perceived positively, with 45.0% of respondents strongly agreeing and 52.3% agreeing on their acceptability. Despite the potential side effects, ART treatment acceptance was high, with 62.5% agreeing and 30% strongly agreeing on its suitability. The SD, ranging from 0.678 to 0.809 across these aspects, indicates moderate variability in perceptions, suggesting a broadly positive reception, but with room for individual differences in experiences. These results reflect that medical professionals' attitudes and possible adverse effects of antiretroviral medications are not perceived to be barriers by KPLHIV.

Table 4 reports the response when KPLHIV were asked about the most common challenges in assessing ART services. These were the distance to medical facilities (21.7%), problems with transportation (10.7%), financial limitations (9.1%), and stigma (6.7%). These results highlight the complexity of the adherence barriers that KPLHIV face and the significance of customized interventions to address these issues and improve medication adherence rates.

**Table 4:** Challenges KPLHIV have faced in accessing ART services ART: Antiretroviral therapy; KPLHIV: Key population people living with HIV

Challenges Assessing ART Services	Frequency	Percentage (%)
Complicated life	1	0.3
Depression	6	1.6
Distance barrier	81	21.7
Family effect	1	0.3
Fear of the unknown	4	1.1
Financial barrier (funds)	34	9.1
Forgetfulness	6	1.6
Insecurity	1	0.3
Lack of psychosocial support	2	0.5
Medication adverse effect	5	1.3
None	160	42.9
Pill burden	3	0.8
Stigma	25	6.7
Transportation	40	10.7
Unemployment	4	1.1
Grand Total	373	100.0

Table 5 shows some correlations between various independent variables and KPLHIV responses on the accessibility of ART services. A Pearson chi-square test with a p-value < 0.05 revealed a highly significant correlation between gender and ART accessibility. The highly significant correlation between gender and ART accessibility suggests that gender identity plays a critical role in influencing access to healthcare services among people living with HIV. This implies that barriers to healthcare access and treatment among PLHIV could be influenced by gender discrimination and inequality among PLHIV. Additionally, access to ART by gender identities may be negatively impacted by various sociocultural factors, including discrimination, stigma, and cultural expectations. There is a significant relationship between age, education level, and marital status of people living with HIV and accessibility to ART in this study, which highlights the multifaceted nature of socioeconomic influences on ART accessibility wherein p-values < 0.05 were found to be significant. This shows that the older population living with HIV may face various ART accessibility barriers possibly due to mobility issues or limited social support networks from stakeholders. Lower educational status might influence reduced health literacy or poor awareness of available services. Marital status also affects access to ART through changes in social support structures or financial resources for an individual. Notably, the employment status of KPLHIV in this study exhibited a highly significant relationship with ART accessibility (p-value < 0.05). On the other hand, the linear-by-linear association test revealed a significant linear trend between employment status and ART accessibility (p-value = 0.049).

**Table 5:** Pearson chi-square test of the association between independent variables (gender, age, marital status, education, employment) and accessibility of ART services df: Degrees of freedom; ^a ^denotes statistically significant results ART: Antiretroviral therapy

Variable	Value	df	Asymptotic Significance (2-Sided)
Gender			
Pearson chi-square	76.384^a^	8	<0.0001
Likelihood ratio	82.239	8	<0.0001
Linear-by-linear association	12.824	1	<0.0001
Number of valid cases	373	-	-
Age at last birthday and accessibility			
Pearson chi-square	471.281^a^	336	<0.001
Likelihood ratio	328.776	336	0.601
Linear-by-linear association	19.242	1	<0.0001
Number of valid cases	373	-	-
Client typology			
Pearson chi-square	207.729^a^	32	<0.0001
Likelihood ratio	192.383	32	<0.0001
Linear-by-linear association	26.143	1	<0.0001
Number of valid cases	373	-	-
Education			
Pearson chi-square	61.628^a^	24	<0.0001
Likelihood ratio	64.679	24	<0.0001
Linear-by-linear association	0.307	1	0.580
Number of valid cases	373	-	-
Marital Status			
Pearson chi-square	61.628^a^	24	<0.0001
Likelihood ratio	64.679	24	<0.0001
Linear-by-linear association	0.307	1	0.580
Number of valid cases	373	-	-
Employment			
Pearson chi-square	133.713^a^	32	<0.0001
Likelihood ratio	132.332	32	<0.0001
Linear-by-linear association	3.878	1	0.049
Number of valid cases	373	-	-

The significant linear trend between employment status and ART accessibility indicates a significant relationship in employment that positively impacts access to ART. The relationship may be influenced by several factors, such as financial stability, access to employer-provided health insurance coverage, or autonomy in scheduling appointments and accessing ART services. Unemployment or underemployment may hinder access to ART due to financial constraints or a lack of insurance coverage. These results highlight how critical it is to consider a range of demographic and socioeconomic variables when addressing PLHIV accessibility-related issues. There is no significant linear trend between educational attainment and ART accessibility (p-value = 0.580). The absence of an observable linear trend in the level of education of PLHIV and accessibility to ART implies that this relationship is more complicated and may be influenced by different factors. Although higher education is often associated with better health outcomes, other obstacles could be mitigated, e.g., geographical location, transport infrastructure, or the inefficiencies of healthcare systems.

The logistic regression analysis provides valuable insights into the barriers faced by people living with HIV in accessing ART services. Age, as a significant variable in this study, shows a negative regression coefficient (-0.068), which implies that, as age increases, the likelihood of affordability and accessibility of ART services decreases. The older population living with HIV may face physical limitations, such as mobility issues, which could hinder their ability to access healthcare facilities. Aging KP may also have increased health complications, leading to significant healthcare needs but reduced access to ART services due to logistical challenges or underlying health complications. The regression coefficients for gender (B = 0.121) and education (B = -0.142) are not statistically significant. This suggested that gender and education may not directly influence access to ART services among people living with HIV in this study. It is essential to consider that, while gender and education may not directly impact accessibility, marital status shows a marginally significant effect (B = -0.387, p = 0.112), indicating a possible influence on ART affordability and accessibility. Marital status may affect access to healthcare based on a few factors, such as social support networks and financial resources. However, the marginal significance between marital status and ART accessibility suggests that the association may not be strong enough to draw definitive conclusions due to the small sample size. The logistic regression model's overall significance was validated by the omnibus test of the model, indicating that the model, as a whole, effectively predicts the likelihood of affordability and accessibility of ART services.

## Discussion

We investigated the barriers faced by KPLHIV in southwestern Nigeria in accessing ART. The study findings show high unemployment rates among KPLHIV, despite their high levels of education. This could be driven by various socio-economic factors and systemic barriers, which can collectively limit employment opportunities and impede the affordability and accessibility of ART [18]. The high percentage of completion of at least secondary education (85.2%) suggests that KPLHIV in this study possess certain levels of education, yet they face challenges in securing stable employment. Previous literature highlights why there could be a high rate of unemployment despite an increased educational trend among PLHIV. Özdemir et al. suggest that higher education does not significantly predict employment for PLHIV; socioeconomic characteristics, age, time since diagnosis, wealth status, illicit drug usage, and CD4 cell count all have an impact on PLHIV employability status [19]. Similarly, Kitshoff et al., in an observational study in KwaZulu-Natal, South Africa, suggest that unemployment can be linked to non-adherence to antiretroviral medication among HIV-positive patients in KwaZulu-Natal due to various factors such as depression and financial constraints [20].

Our study findings also reveal that transportation costs pose a significant barrier, with the majority of participants experiencing difficulties in affording transportation to ART clinics. This result confirms previous study findings in Uganda, which highlighted transportation as a significant obstacle to accessing healthcare services, underscoring the importance of tackling this challenge to enhance healthcare access [21]. This may be influenced by socio-economic factors, including limited financial resources and the geographical distribution of healthcare facilities, and can be tackled through the provision of jobs, public transportation, and welfare incentives to KPLHIV [22,23]. Inadequate public transportation infrastructure and long distances to ART clinics in remote areas exacerbate transportation challenges [23]. The result of this study shows that, despite transportation barriers, the perception of service fees at ART facilities was not a significant barrier among PLHIV in this study, which suggests potential discrepancies in the cost burden experienced by KPLHIV. For instance, some respondents in this study attributed their inability to access ART services to a lack of financial resources, citing insufficient funds as the primary reason for their inability to transport themselves to these facilities. This discrepancy may be a result of government subsidies, donor funding, or humanitarian support programs targeted at ART services for PLHIV in Nigeria [24].

Additionally, findings from this study show that there is high satisfaction with the quality of care received at ART clinics, which indicates positive experiences, potentially outweighing financial concerns among PLHIV in this study. This corresponds with recent research in Ethiopia, which estimated the acceptability of ART services among PLHIV to be approximately 75.0%, indicating a generally favorable view of PLHIV towards healthcare provision and treatment modalities [25]. This suggests that, despite the significant financial burden of traveling to the nearest ART clinic, some PLHIV in this study prioritize their health and consistently commute to the clinic due to the exceptional care provided at the clinic. The acceptability of ART services among participants in this study may be influenced by positive perceptions of interactions with medical professionals, privacy measures, and staff attitudes [26]. This study's findings correspond with a study that examined the acceptability of ART, tuberculosis, and maternal health services from patients’ viewpoints in a Johannesburg sub-district, South Africa, which shows that there is significant acceptability of ART due to the perception of the interaction of medical personnel and other related factors. However, persistent challenges in accessing ART services, such as distance to medical facilities, transportation issues, financial limitations, and stigma, underscore the complex and multifaceted nature of adherence barriers faced by KPLHIV [27]. Table 5 in the results section demonstrates the correlations between various factors and accessibility to ART services among KPLHIV. A Pearson chi-square test revealed a highly significant correlation between gender and ART accessibility (p-value < 0.05), suggesting that gender identity plays a critical role in accessing healthcare services. Age, education level, and marital status also showed significant relationships with ART accessibility (p-values < 0.05), highlighting the complex socioeconomic influences on access to ART care. Employment status exhibited a significant relationship with ART accessibility (p-value < 0.05), with a significant linear trend (p-value = 0.049). This may be due to financial stability, health insurance coverage, and autonomy in scheduling appointments, which positively impact access to ART [28]. In contrast, educational attainment did not show a significant linear trend with ART accessibility (p-value = 0.580). These findings emphasize the importance of considering demographic and socioeconomic variables when addressing barriers relating to KPLHIV.

In this study, the logistic regression analysis reveals age as a significant barrier to accessing ART services, with a negative regression coefficient (-0.068). This suggests that, as age increases, the likelihood of affordability and accessibility of ART services decreases. Variably, the older population living with HIV may face physical limitations, such as mobility issues, which could hinder their ability to access healthcare facilities. Aging key populations may also have increased health complications, leading to significant healthcare needs, but reduced access to ART services due to logistical challenges or underlying health complications [29]. Gender (B = 0.121) and education (B = -0.142) show no statistically significant influence on access to ART services. Marital status has a marginally significant effect (B = -0.387, p = 0.112), suggesting a possible impact on ART affordability and accessibility through social support networks and financial resources. However, the association is not strong enough to draw a definitive conclusion. The omnibus test of the model confirms its overall significance, effectively predicting the likelihood of affordability and accessibility of ART services based on demographic and socioeconomic variables.

This study faces limitations due to its reliance on self-reported data, which might introduce biases. Additionally, variations in healthcare systems may limit the applicability of the findings, with their relevance potentially confined to the regions investigated. Further research is needed to delve into the underlying causes of the high levels of satisfaction with healthcare services and the positive perceptions of healthcare worker attitudes observed in this study.

## Conclusions

This study highlights the barriers faced by KPLHIV in southwestern Nigeria in accessing ART services. Despite high levels of education, unemployment rates remain high, possibly driven by socio-economic factors and systemic barriers. Unemployment, transportation costs, and lack of funds posed substantial barriers among KPLHIV in southwestern Nigeria, underscoring the need for targeted interventions to address these challenges.

These barriers are, however, tempered by the high satisfaction with the quality of healthcare services and healthcare workers’ attitudes reported by this subpopulation. To improve access to ART services, tailored interventions are necessary, focusing on addressing systemic barriers, improving transportation infrastructure, and providing financial assistance. Community-based initiatives and peer support networks can also play a crucial role in addressing some of the barriers.
